# Why teaching innovation matters: Evidence from a pre- versus peri-COVID-19 pandemic comparison of student evaluation data

**DOI:** 10.3389/fpsyg.2022.963953

**Published:** 2022-08-17

**Authors:** Pei-Hsin Lin, Lee-Rong Huang, Sheng-Hsiang Lin

**Affiliations:** ^1^Office of Research and Development, National Cheng Kung University, Tainan, Taiwan; ^2^Office of Strategic Planning, National Cheng Kung University, Tainan, Taiwan; ^3^Institute of Clinical Medicine, College of Medicine, National Cheng Kung University, Tainan, Taiwan; ^4^Department of Public Health, College of Medicine, National Cheng Kung University, Tainan, Taiwan; ^5^Biostatistics Consulting Center, National Cheng Kung University Hospital, College of Medicine, National Cheng Kung University, Tainan, Taiwan

**Keywords:** teaching innovation, higher education, teacher development, student evaluation of teaching, COVID-19 pandemic

## Abstract

The COVID-19 pandemic has robustly affected global education environments, so higher education institutions need to emphasize innovation and creativity in educational methods for teachers to improve their teaching performance as well as enhance the engagement and motivation of students in this changing environment. Accordingly, it is essential to discuss the role of teaching innovation in the setting of the COVID-19 pandemic compared to the pre-COVID-19 period. The aim of this study is to empirically validate the importance of teaching innovation in student evaluation of teaching before and during the COVID-19 pandemic. Data were collected from the medical college of a comprehensive university in Taiwan and were subjected to *t*-tests and multiple linear regression analysis. Findings from a quantitative study with 44 teachers revealed that teaching innovation was positively correlated to student evaluation of teaching. We also found that teachers who implemented teaching innovation strategies performed better than those teachers who used conventional teaching strategies on student evaluation of teaching. In particular, teaching innovative teachers had improvement in student evaluation of teaching during the time of the COVID-19 pandemic, but not non-teaching innovative teachers. The evidence from this study suggests that teaching innovation can not only enhance teachers’ teaching development and performance but also boost students’ motivation for learning, especially in the time of the COVID-19 pandemic. Our findings have important implications for future research on teaching innovation and for higher education institutions and faculty wishing to provide high-quality learning environments to their students.

## Introduction

The COVID-19 pandemic has had a great impact on the economic, social, and political environments globally, and the educational environment is no exception (Aristovnik et al., 2020; Daniel, 2020). Due to the national lockdown and social distancing measures, higher education institutions have been forced to undergo significant transformation, adopt innovative ways of remote teaching and learning using up-to-date digital technologies for knowledge delivery, and renew their business models in order to adapt to difficulties and challenges posed by the ongoing COVID-19 pandemic (Albers-Miller et al., 2001; Dwivedi et al., 2020; Toquero, 2020; Bygstad et al., 2022). As a result, many higher education units have encouraged their faculty to improve their teaching performance through educational innovations (Yin et al., 2017; Cao et al., 2020; Chand et al., 2020). That is, the higher education systems across the world need to invest in the professional development of teachers, especially effective pedagogy and information and communications technology, considering the present COVID-19 scenario. Making teaching creative, innovative, and interactive through user-friendly tools is a new field of research and development (Neuwirth et al., 2021; Pokhrel and Chhetri, 2021). Therefore, the COVID-19 crisis provides an opportunity to reflect on the idea that teaching innovation may be necessary for sustainable teacher professional development in the future (Petrila et al., 2022).

A lesson learnt from the COVID-19 pandemic is that teachers should be oriented towards the use of innovative teaching methods and tools. So far, however, most previous studies of teaching innovation have dealt with teachers’ innovative performance by using a teacher innovative work behavior questionnaire (Zhang and Zhang, 2012; Thurlings et al., 2015; Li et al., 2017; Yu et al., 2021), whereas the use of student evaluation of teaching (SET) has not been investigated. In addition, recently investigators have examined the effects of the pandemic on university students’ engagement, learning environments, and mental health and wellness by comparing analytics data during both pre- and peri-COVID-19 pandemic (Copeland et al., 2021; Long et al., 2022; Summers et al., 2022), however, there have been no comparative studies which have identified differences in teaching innovation performance between pre- and peri-COVID-19 pandemic surveys. Therefore, focusing on higher education, this study aims to highlight the importance of teaching innovation and how teaching innovation contributes to student evaluation by considering both pre- and peri-COVID-19 pandemic data. The present study contributes to higher education literature by providing further evidence of how teaching innovation can benefit educators’ teaching performance from the students’ perspective and generates fresh insights into teaching innovation issues as higher education institutions and faculty prepare themselves for the post-COVID-19 era.

### Teaching innovation and student evaluation

An innovative teacher would be willing to explore up-to-date and diverse strategies and approaches in teaching practices in order to sustain students’ learning interest and motivation, enhance the academic performance of the students, and add value to students’ learning procedure (Lim et al., 2011; Nemeržitski et al., 2013; Zhu et al., 2013). To date, a large and growing body of literature has investigated the key competencies and characteristics of teaching innovation (Horng et al., 2005; Zhu et al., 2013; Zhu and Wang, 2014; Tan et al., 2022). One study by Jaskyte et al. (2009) explored a number of factors affecting teaching innovation from both students’ and teachers’ points of view, such as teaching style (e.g., enthusiasm and engagement), classroom culture (e.g., flexible and creative response to content and student needs), teacher-student relationships (e.g., interpersonal interactions and communications), and teaching methods/strategies (e.g., unorthodox assessment tools, materials and assignments). In particular, flexible learning environments (Willems, 2005; Collis and Moonen, 2012; Joan, 2013) and teacher–student interaction (Lynch, 2001; Zhu et al., 2013; Pennings et al., 2018), which are student-centered educational practices, help to promote quality education. Additionally, previous research has established that organizational abilities can affect the success of teaching innovations and teaching and learning effectiveness (Anderson et al., 2008; Renta-Davids et al., 2016). Moreover, based on expectancy-value theory, student perceptions of task values (interest, usefulness, and importance) are related to teachers’ pedagogical practices (Neuville et al., 2007; Chiu and Wang, 2008). Specifically, intrinsic value (perception of interest) is most related to innovative teaching and learning issues as teachers have to draw students’ interest and attention in innovative ways (Simplicio, 2000; Morris and Chikwa, 2014; Cevikbas and Argün, 2017; Fiksl et al., 2017).

Several lines of evidence suggest that students are viewed as customers in higher education (Guilbault, 2016; Calma and Dickson-Deane, 2020; Raza et al., 2021). A customer-oriented higher education institution collects and acts on student feedback on teaching and other related processes, and values students’ voices as one of the criteria for teacher appraisal (Koris et al., 2015; Mazandarani and Troudi, 2022). As teaching innovation is a reciprocal process, students’ opinions on courses and teaching also influence the way teachers teach (Johnson, 2000; Denson et al., 2010; Hornstein, 2017). Nowadays, student evaluation of teaching (SET) has become a primary means used by higher education institutions for the purpose of improving teaching and learning (Ballantyne et al., 2000; Ardalan et al., 2007; Tucker, 2014; Debroy et al., 2019; Cook et al., 2022). It is now well established by a variety of studies that results obtained from SET help universities to assess faculty’s teaching quality and provide teaching staff with insights into the strengths and weaknesses of their teaching practice (Richardson, 2005; Balam and Shannon, 2010; Alderman et al., 2012; Palmer, 2012; Estelami, 2015; McClain et al., 2018; Hoel and Dahl, 2019; Samuel, 2021). Taken together, it seems that we must pay attention to not only the effects of teaching innovation but also multi-dimensional SET measures. In the current study, the multidimensional SET survey encompasses nine factors that are necessary for teachers to be innovative in their educational activities, including teacher enthusiasm, organizational skills, flexible teaching, content coverage, teacher-student interaction, interpersonal harmony, assessment method, homework assignment, and intrinsic learning value, and the sample was separated into teaching innovation and non-teaching innovation groups to investigate the important role of teaching innovation in the higher education context.

### Research questions

After performing the above-mentioned review of the literature regarding teaching innovation and SET, we formulated three research questions. Research question 1 is concerned with changes in SET before and during the COVID-19 pandemic. Research question 2 is focused on the teaching innovation gap by comparing different sample groups. Research questions 3 was carried out to investigate the relationship between teaching innovation and SET.

• Research question 1: Are there differences in the student evaluation of teaching in the pre- and peri-COVID-19 pandemic periods?
• Research question 2: Are there differences in the student evaluation of teaching of the teaching innovation and non-teaching innovation groups?
• Research question 3: Does teaching innovation predict student evaluation in the teaching pre- and peri-COVID-19 pandemic periods?

## Methods

### Study design and data collection

Data were collected at the medical college of a comprehensive university in Taiwan. COVID-19 (coronavirus disease 2019) was discovered in December 2019. In order to achieve a viable comparison of student evaluation between the pre- and peri-COVID-19 pandemic periods, we gathered end-of-semester student evaluation data over the past three years. Data collection for the pre-COVID-19 pandemic period took place from September 2018 to January 2020, and for the peri-COVID-19 pandemic period it took place from February 2020 to June 2021. To identify the teaching innovation group, we collected the lists of 2019/2020/2021 “Teaching Innovation Award” winners. Among the 300 teaching faculty, there were 22 who were award winners (i.e., the teaching innovation group). Then a sample of 22 medical teaching faculty who were not award winners were chosen by using the random sampling method as the non-teaching innovation group. The study was set in Taiwan.

### Measures

To collect teachers’ demographic details regarding gender, age, designation, and teacher affiliation, the higher education database was employed. The online questionnaire-based survey was conducted to access the end-of-semester SET survey. The survey asked students to complete nine questions that asked about teacher enthusiasm, organizational skills, flexible teaching, content coverage, teacher-student interaction, interpersonal harmony, assessment method, reading and homework, and intrinsic learning value. Each question was cast on a 5-point Likert scale, ranging from 1 (Disagree strongly) to 5 (Agree strongly). The operational definitions and items for each dimension were as follows:

• Teacher enthusiasm: the conjoined occurrence of enjoyment and behavioral expression (Keller et al., 2016). The item for this dimension is “I think the instructor is enthusiastic and conscientious.”
• Organizational abilities: skills such as time management and systematic teaching method that the teacher applies for enhancing the effectiveness of teaching and learning (Anderson et al., 2008). The item for this dimension is “I think the instructor is organized and able to instruct in a systematic way.”
• Flexible teaching: flexibility for learners by anticipating and responding to their ever-changing needs and expectations (Willems, 2005). The item for this dimension is “I think the instructor pays attention to students’ responses and can properly adjust the pace of teaching according to students’ demands.”
• Content coverage: the depth (a broad range of subjects around a given focus topic) and breadth (the full span of knowledge of a subject) of the curriculum (Schwartz et al., 2009). The item for this dimension is “I think the instructor is well prepared for the course content and the content is applicable.”
• Teacher-student interaction: daily interpersonal interactions and effective communication in the classroom (Pennings et al., 2018). The item for this dimension is “I think the instructor encourages discussion and asks questions in class.”
• Interpersonal harmony: teacher and student concurrently sense tangible coordination, cooperation, and congeniality (Veldman et al., 2013). The item for this dimension is “I think the instructor treats students’ questions and feedback diligently.”
• Assessment methods: methods teachers use when introducing more realistic and meaningful tasks and providing broader and more reliable indicators of students’ achievements (e.g., case studies and group collaboration projects) (Rust, 2002). The item for this dimension is “I think the instructor giving examinations or feedback on reports contributes to my learning.”
• Homework assignments: teachers’ use of assignments for the purpose of practicing and reviewing the material taught in class (Rosário et al., 2015). The item for this dimension is “I think the instructor’s reading list or assignments are helpful for students’ learning.”
• Intrinsic learning value: students’ emotional attachment and enjoyment of engaging in the activity or task (Eccles, 2009). The item for this dimension is “I think the instructor’s way of teaching inspires my creative thinking and learning interest.”

### Data analysis

All statistical analyses draw on data from a total of 44 teaching faculty working in the college of medicine. Considering that the sampling method of this study was random sampling, the chi-square test of homogeneity was used to evaluate whether the distribution of a variable differs across two or more groups. The paired samples *t*-test was carried out to examine the difference in SET in the pre- and peri-COVID-19 pandemic periods (RQ1). To compare the difference between the teaching innovation and non-teaching innovation groups, the independent samples *t*-test was applied (RQ2). The multiple regression analysis was carried out to answer RQ3 for investigating the relation between teaching innovation and SET. Since confounding factors are the major concerns in causal studies, gender, age, designation, and teacher affiliation were considered to be the potential confounders in the design of this study. SAS (version 9.4) was utilized for the purpose of data analysis. Significance (p-value) were set at the 95% confidence level. We used Cronbach’s alpha to estimate the reliability of the survey instrument. The Cronbach’s α coefficient was 0.992, which had good internal consistency.

## Results

### Demographic characteristics

The chi-square test was utilized to determine whether the data were from the same population or were homogeneous. Table 1 exhibits the sample characteristics and results of the homogeneity test, revealing that all *p*-values were greater than the significance level of 0.05. Thus, it can be said that the samples come from the homogeneous population. Of the 44 medical teachers, 23 (52%) were female and 21 (48%) were male. About half of them (59%) were between 41 and 50 years old. In terms of designation, there were 14 (32%) professors, nine (41%) associate professors, and 12 (27%) assistant professors, while 70% of the sample of teachers served in the department and the remainder, 30%, served in the institute.

**TABLE 1:** Sample characteristics for the teaching innovation and non-teaching innovation groups.

Variables	Characteristic	Total	Teaching innovation (*n* = 22)		Non-Teaching innovation (*n* = 22)		*p*-value
				
			n	%	n	%	
Gender	Male	21	7	31.8	14	63.6	0.069
	Female	23	15	62.2	8	36.4	
Age	35–40	4	1	4.50	3	13.6	0.399
	41–45	12	7	31.8	5	22.7	
	46–50	14	9	40.9	5	22.7	
	51–55	4	2	9.10	2	9.10	
	56–60	4	2	9.10	2	9.10	
	> 60	6	1	4.50	5	22.7	
Designation	Assistant Professor	12	6	27.3	6	27.3	1.000
	Associate Professor	18	9	40.9	9	40.9	
	Professor	14	7	31.8	7	31.8	
Teacher affiliation	Department	31	16	72.7	15	68.2	0.741
	Institute	13	6	27.3	7	31.8	

### Differences in student evaluation of teaching in the pre- and peri-COVID-19 pandemic periods

The paired samples *t*-test was performed to compare student evaluation in the pre- and peri-COVID-19 pandemic periods. The data were analyzed and the results are displayed in Table 2. As Table 2 shows, for the teaching innovation group, the results indicate that there were significant differences in teacher enthusiasm, organizational abilities, flexible teaching, content coverage, teacher-student interaction, interpersonal harmony, and overall student evaluation in the pre- and peri-COVID-19 pandemic periods. On the other hand, the non-teaching innovative group did not show any significant differences in student evaluation in the two periods. Therefore, teaching innovative educators had a significantly greater student evaluation regarding teacher enthusiasm, organizational abilities, flexible teaching, content coverage, teacher-student interaction, and interpersonal harmony during the COVID-19 pandemic than before the pandemic.

**TABLE 2:** Differences in SET in the pre- and peri-COVID-19 pandemic periods.

Student evaluation of teaching	Variables	Teaching innovation (*n* = 22)			Non-Teaching innovation (*n* = 22)		
		Mean	*SD*	*p*-value	Mean	*SD*	*p*-value
Teacher enthusiasm	Pre-COVID-19 pandemic	4.592	0.163	0.016*	4.389	0.180	0.906
	Peri-COVID-19 pandemic	4.647	0.173		4.394	0.163	
Organizational abilities	Pre-COVID-19 pandemic	4.539	0.178	0.043*	4.272	0.279	0.904
	Peri-COVID-19 pandemic	4.593	0.200		4.278	0.212	
Flexible teaching	Pre-COVID-19 pandemic	4.504	0.168	0.003**	4.280	0.256	0.973
	Peri-COVID-19 pandemic	4.577	0.194		4.278	0.214	
Content coverage	Pre-COVID-19 pandemic	4.530	0.185	0.009**	4.339	0.217	0.759
	Peri-COVID-19 pandemic	4.599	0.198		4.353	0.191	
Teacher-student interaction	Pre-COVID-19 pandemic	4.553	0.154	0.036*	4.332	0.186	0.946
	Peri-COVID-19 pandemic	4.618	0.169		4.335	0.171	
Interpersonal harmony	Pre-COVID-19 pandemic	4.577	0.143	0.049*	4.354	0.201	0.888
	Peri-COVID-19 pandemic	4.635	0.168		4.348	0.182	
Assessment methods	Pre-COVID-19 pandemic	4.498	0.186	0.666	4.322	0.223	0.437
	Peri-COVID-19 pandemic	4.512	0.218		4.281	0.197	
Homework assignments	Pre-COVID-19 pandemic	4.499	0.181	0.292	4.324	0.199	0.337
	Peri-COVID-19 pandemic	4.529	0.207		4.276	0.178	
Intrinsic learning value	Pre-COVID-19 pandemic	4.509	0.171	0.127	4.287	0.248	0.485
	Peri-COVID-19 pandemic	4.561	0.192		4.250	0.179	
Overall	Pre-COVID-19 pandemic	4.533	0.162	0.041*	4.322	0.216	0.801
	Peri-COVID-19 pandemic	4.586	0.185		4.310	0.176	

*p < 0.05, **p < 0.01.

### Differences in student evaluation of teaching in the teaching innovation and non-teaching innovation groups

To compare the difference between the teaching innovation and non-teaching innovation groups, the independent samples *t*-test was utilized. Table 3 presents the summary statistics for the independent samples *t*-test. The results, as shown in Table 3, indicate that the difference between the teaching innovation and non-teaching innovation groups was significant, revealing that the teaching innovation group performed significantly better on student evaluation regarding teacher enthusiasm, organizational abilities, flexible teaching, content coverage, teacher-student interaction, interpersonal harmony, assessment methods, homework assignments, and intrinsic learning value than the non-teaching innovation group, regardless of the COVID-19 pandemic.

**TABLE 3:** Differences in SET of the teaching innovation and non-teaching innovation groups.

Student evaluation of teaching	Variables	Pre-COVID-19 pandemic			Peri-COVID-19 pandemic		
		Mean	*SD*	*p*-value	Mean	*SD*	*p*-value
Teacher enthusiasm	Teaching innovation	4.592	0.163	< 0.001***	4.647	0.173	< 0.001***
	Non-Teaching innovation	4.389	0.180		4.394	0.163	
Organizational abilities	Teaching innovation	4.539	0.178	< 0.001***	4.593	0.200	< 0.001***
	Non-Teaching innovation	4.272	0.279		4.278	0.212	
Flexible teaching	Teaching innovation	4.504	0.168	0.001**	4.577	0.194	< 0.001***
	Non-Teaching innovation	4.280	0.256		4.278	0.214	
Content coverage	Teaching innovation	4.530	0.185	0.003**	4.599	0.198	< 0.001***
	Non-Teaching innovation	4.339	0.217		4.353	0.191	
Teacher-student interaction	Teaching innovation	4.553	0.154	< 0.001***	4.618	0.169	< 0.001***
	Non-Teaching innovation	4.332	0.186		4.335	0.171	
Interpersonal harmony	Teaching innovation	4.577	0.143	< 0.001***	4.635	0.168	< 0.001***
	Non-Teaching innovation	4.354	0.201		4.348	0.182	
Assessment methods	Teaching innovation	4.498	0.186	0.007**	4.512	0.218	0.001**
	Non-Teaching innovation	4.322	0.223		4.281	0.197	
Homework assignments	Teaching innovation	4.499	0.181	0.004**	4.529	0.207	< 0.001***
	Non-Teaching innovation	4.324	0.199		4.276	0.178	
Intrinsic learning value	Teaching innovation	4.509	0.171	0.001**	4.561	0.192	< 0.001***
	Non-Teaching innovation	4.287	0.248		4.250	0.179	
Overall	Teaching innovation	4.533	0.162	0.001**	4.586	0.185	< 0.001***
	Non-Teaching innovation	4.322	0.216		4.310	0.176	

**p < 0.01, ***p < 0.001.

### Associations between teaching innovation and student evaluation

The multiple linear regression analysis was conducted to further examine the associations between teaching innovation and SET. Simultaneously, we adjusted the confounders, including gender, age, designation, and teacher affiliation, in order to prevent distortion of the results. The results of the multiple linear regression analysis for student evaluation before the COVID-19 pandemic are set out in Table 4. The results demonstrated that teaching innovation (β = 0.528, *p* < 0.001) was significantly associated with overall student evaluation before the COVID-19 pandemic, and explained 29.9% of overall student evaluation. The results of the multiple linear regression analysis for student evaluation during the COVID-19 pandemic are set out in Table 5. The results demonstrated that teaching innovation (β = 0.575, *p* < 0.001) was significantly associated with overall student evaluation during the COVID-19 pandemic, and explained 39.4% of overall student evaluation. By considering the potential confounding factors (gender, age, designation, and teacher affiliation) during analysis, we found that confounders could not bias this study results, providing the study’s statistical precision. Together these results provide important insights into the positive influence of teaching innovation on student evaluation. It is clear to note that the amount of explained variance for the peri-COVID-19 pandemic period was higher than for the pre-COVID-19 pandemic period, implying that teaching innovation was more important than ever before for educators to improve their SET scores.

**TABLE 4:** Associations between teaching innovation and SET in the pre-COVID-19 pandemic period.

Student evaluation of teaching	Predictor: Teaching innovation			Confounders	R^2^	Adj.R^2^
	Standardized Coefficients(β)	*t*-value	*p*-value			
Teacher enthusiasm	0.541	4.085	< 0.001***	n.s.	0.414	0.336
Organizational abilities	0.514	3.790	= 0.001**	n.s.	0.387	0.307
Flexible teaching	0.525	3.891	< 0.001***	n.s.	0.393	0.313
Content coverage	0.461	3.276	= 0.002**	n.s.	0.338	0.251
Teacher-student interaction	0.563	4.302	< 0.001***	n.s.	0.429	0.354
Interpersonal harmony	0.607	4.708	< 0.001***	n.s.	0.446	0.373
Assessment methods	0.457	3.151	= 0.003**	n.s.	0.297	0.205
Homework assignments	0.463	3.243	= 0.002**	n.s.	0.320	0.231
Intrinsic learning value	0.504	3.542	= 0.001**	n.s.	0.324	0.235
Overall	0.528	3.872	< 0.001***	n.s.	0.381	0.299

**p < 0.01, ***p < 0.001, n.s. = no significance, we adjusted for the effect of the confounders (gender, age, designation, and teacher affiliation) in the analyses.

**TABLE 5:** Associations between teaching innovation and SET in the peri-COVID-19 pandemic period.

Student evaluation of teaching	Predictor: Teaching innovation			Confounders	R^2^	Adj.R^2^
	Standardized Coefficients(β)	*t*-value	*p*-value			
Teacher enthusiasm	0.590	4.512	< 0.001***	n.s.	0.425	0.350
Organizational abilities	0.578	4.682	< 0.001***	n.s.	0.487	0.419
Flexible teaching	0.574	4.542	< 0.001***	n.s.	0.462	0.392
Content coverage	0.493	3.645	= 0.001**	n.s.	0.384	0.303
Teacher-student interaction	0.610	4.991	< 0.001***	n.s.	0.494	0.432
Interpersonal harmony	0.601	4.863	< 0.001***	n.s.	0.486	0.419
Assessment methods	0.453	3.260	= 0.002**	n.s.	0.352	0.267
Homework assignments	0.510	3.778	= 0.001**	n.s.	0.386	0.305
Intrinsic learning value	0.607	4.949	< 0.001***	n.s.	0.498	0.427
Overall	0.575	4.557	< 0.001***	n.s.	0.465	0.394

**p < 0.01, ***p < 0.001, n.s. = no significance, we adjusted for the effect of the confounders (gender, age, designation, and teacher affiliation) in the analyses.

## Discussion

The purpose of this research was to determine the effect of teaching innovation by comparing SET data before and during the time of the COVID-19 pandemic in a higher education context. In this section, we discuss the results in relation to our three research questions. With respect to the first research question, we found that overall SET scores increased among the teaching innovation group. During COVID-19, the teaching innovative teachers were capable of improving their SET scores for teacher enthusiasm, organizational abilities, flexible teaching, content coverage, teacher-student interaction, and interpersonal harmony. However, the non-teaching innovative teachers showed no improvements in their SET scores during the time of the COVID-19 pandemic. The second research question focused on the differences in the SET of the teaching innovative and non-teaching innovative teachers. The results showed that teaching innovative teachers had a higher degree of SET scores than non-teaching innovative teachers, particularly, the most significant differences were observed for organizational abilities and intrinsic learning value. Taken together, these results confirm that teaching innovation has a positive influence on their teaching quality and performance. These results match those observed in earlier studies. For example, Fiksl et al. (2017) found that innovative pedagogies are effective in terms of fostering students’ creative thinking and learning motivation.

With respect to the last research question, results from the multiple linear regression analysis demonstrated that teaching innovation was positively relevant to all aspects of SET scores, regardless of the COVID-19 pandemic. We also found that the predictive power of teaching innovation for SET increased in the peri-COVID-19 scenario compared with the pre-COVID-19 pandemic period, and the most interesting finding was that there was a remarkable increase in the intrinsic learning value. Hence, the evidence from this study suggests that teaching innovation can not only enhance educators’ teaching development and performance but can also boost students’ motivation for learning, especially in the time of the COVID-19 pandemic. These results are in accordance with recent studies indicating that the COVID-19 pandemic required teaching faculty to adapt to a rapid transition from traditional instruction to innovative teaching and learning formats (Neuwirth et al., 2021; Pokhrel and Chhetri, 2021; Petrila et al., 2022). Therefore, providing innovative teaching and learning practices during a crisis can be seen as a tool for instilling greater intrinsic motivation, enjoyment, and vitality amongst students.

## Conclusion and implications

This study set out to highlight the importance of teaching innovation by investigating the changes in SET before and during the COVID-19 pandemic as well as the relationships between educators’ teaching innovation and student evaluation in the field of higher education. The principal theoretical implication of this study is that we confirmed that teaching innovation plays an important role in teaching quality and student learning value after the unexpected transition due to the COVID-19 pandemic. Before this study, evidence of teaching innovation after the COVID-19 outbreak was purely anecdotal. The present study appears to be the first comparative study to determine the effectiveness of teaching innovation in the COVID-19 pandemic setting and certainly adds to our understanding of innovative teaching strategies. This study has two main practical implications. First, it provides a deeper insight into how students assess teacher innovations in the setting of the COVID-19 pandemic. From students’ points of view, teacher enthusiasm, flexible teaching, teacher-student interaction, and interpersonal harmony were most related to innovative teaching strategies. Thus, an innovative teacher can motivate a student through enthusiasm, flexible classroom culture, and interpersonal interactions and communications in times of COVID-19. Secondly, university instructors should be ready to provide innovative teaching methods after the COVID-19 outbreak, which will lead to high teaching effectiveness and levels of student learning value and engagement. Therefore, it seems that higher education institutions should provide more opportunities for capacity development and pedagogical innovations to help teachers improve both the quality of their teaching and their effectiveness when promoting high quality education.

## Limitations and future directions

The generalizability of these results is subject to certain limitations. First, in terms of sample population and size, the samples of this study were drawn from a college of medicine and the small sample size could lead to bias. For future studies, a larger sample size is required to improve the generalizability of the research findings. Second, this study is limited by the lack of qualitative data. Further research should employ qualitative techniques such as an open-ended survey questionnaire and interviews to investigate students’ opinions on teaching performance and effectiveness. Thirdly, this study was conducted in Taiwan. Further research could be conducted in other countries to extend the understanding of teaching innovation from a global perspective. Finally, recent literature has emerged that offers contradictory findings about the value of SET. For instance, Lawrence (2018) argued that SET scores are imperfect measures of instructor performance and likely undermine educational standards. Kreitzer and Sweet-Cushman (2021) found that SETs have low or no correlation with learning, and women faculty, faculty of color, and other marginalized groups are subject to a disadvantage in SETs. Therefore, further work should be cautioned about the use of SETs in appraisal, and alternatives assessments of teaching should be further utilized such as peer evaluation and external/internal observation. Although the current study is based on a small sample of participants, the findings suggest that teaching innovation is the new normal since the COVID-19 pandemic. Higher education institutions should support and promote innovative methods of teaching strategies for improving teacher performance and enhancing the student learning process.

## Data availability statement

The original contributions presented in the study are included in the article/supplementary material, further inquiries can be directed to the corresponding author/s.

## Ethics statement

Ethical review and approval were not required for the study on human participants in accordance with the local legislation and institutional requirements. Written informed consent for participation was not required for this study in accordance with the national legislation and the institutional requirements.

## Author contributions

P-HL and S-HL contributed to the conception and design of the study. P-HL contributed to the writing of the full manuscript and performed the statistical analysis. S-HL provided critical revisions and supervised the manuscript. L-RH organized the database. All authors approved the final version of the manuscript.
